# Supplementary material for: Characterization of Enrichment Cultures of Anammox, Nitrifying and Denitrifying Bacteria Obtained from a Cold, Heavily Nitrogen-Polluted Aquifer
Source: Biology (Basel). 2023 Jan 30;12(2):221. doi: 10.3390/biology12020221 (PMC9952944; doi:10.3390/biology12020221)
Supplement: Supplementary file 1 [file biology-12-00221-s001.zip › biology-2101784-supplementary.pdf]

Supplementary

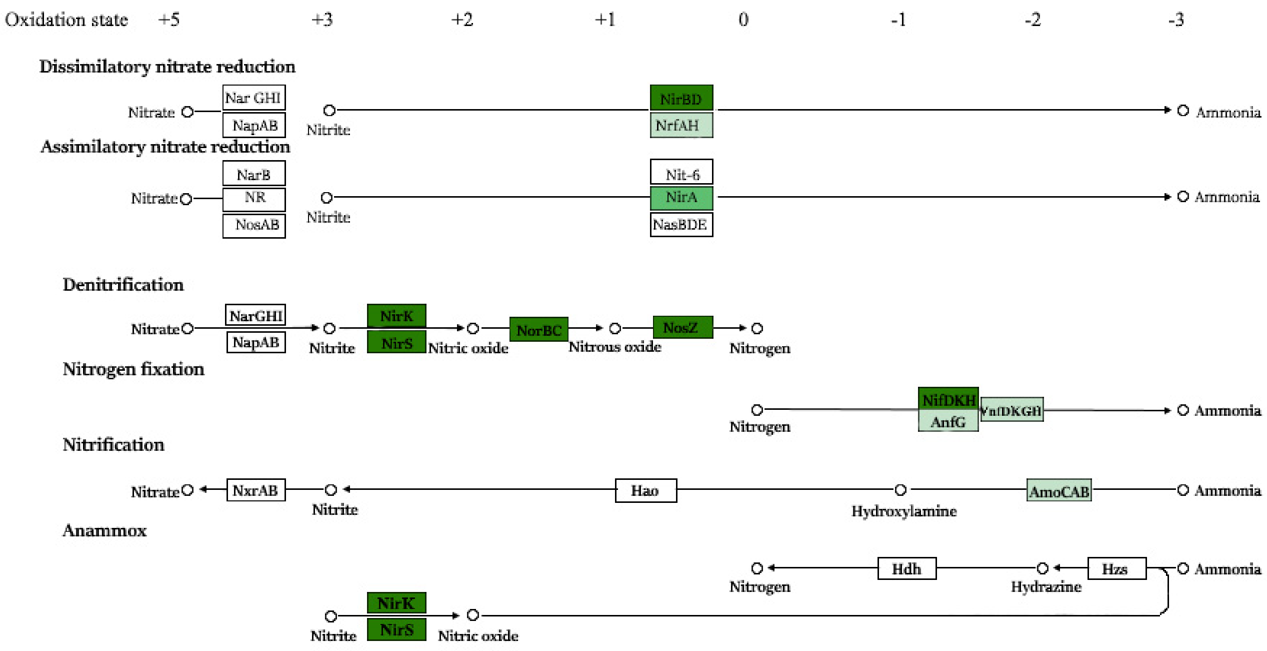

A

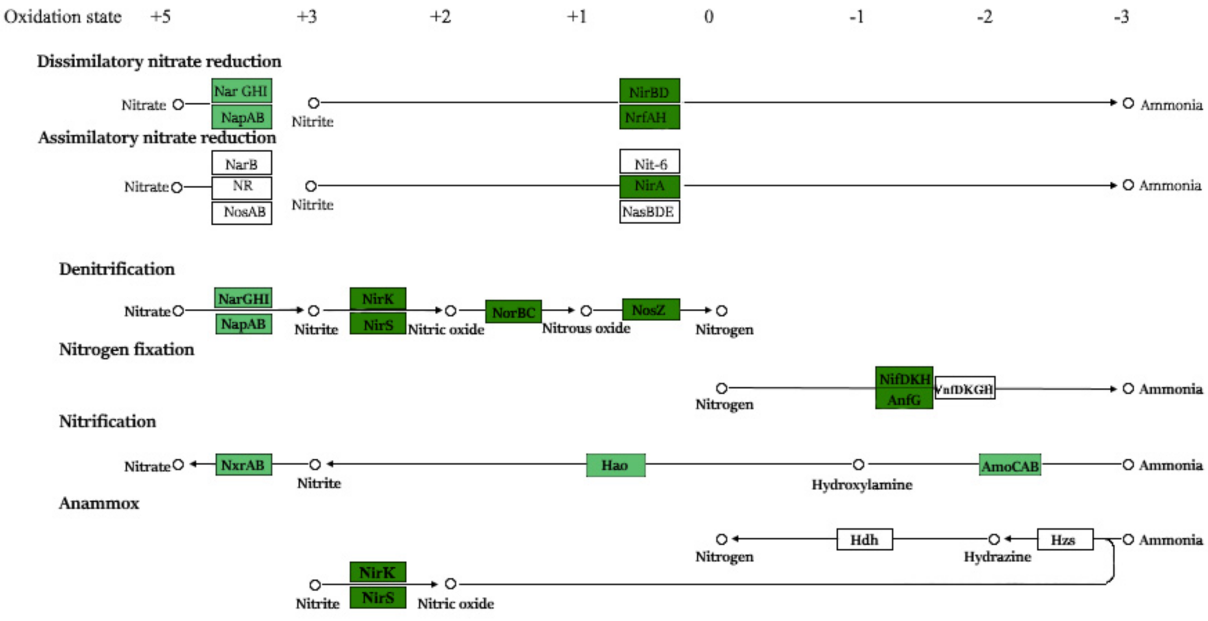

B

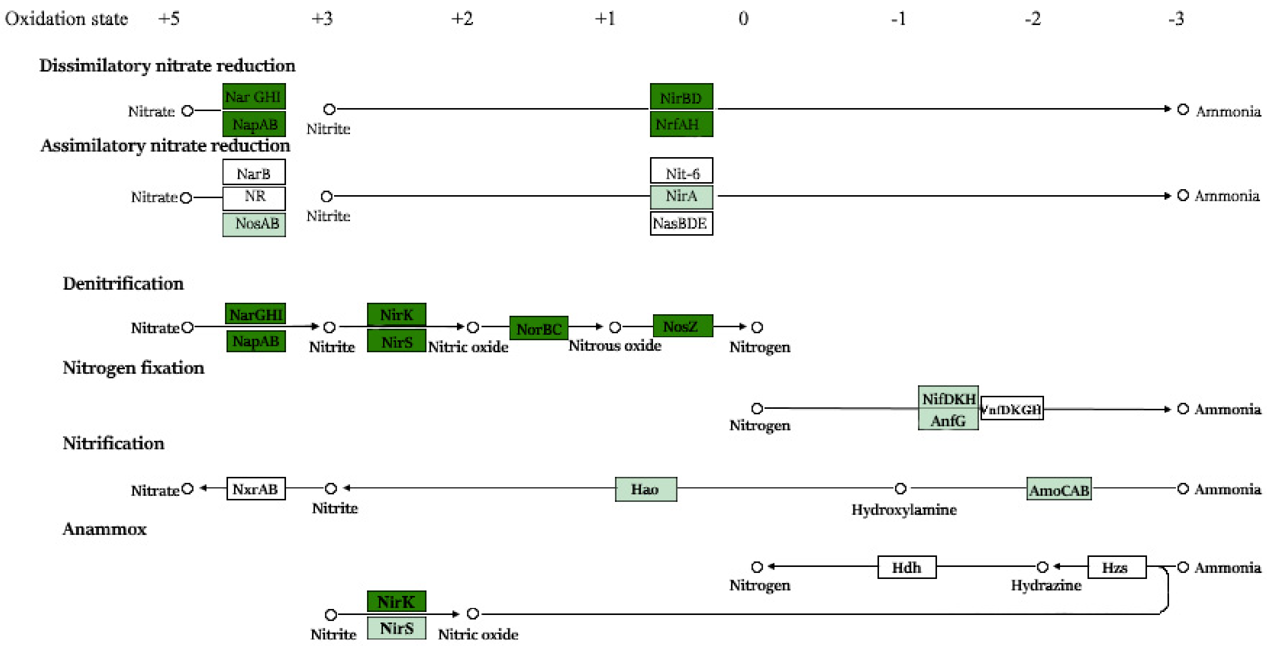

Oxidation state +5 +3 +2 +1 0 -1 -2 -3
Dissimilatory nitrate reduction
Nitrate
Nar GHI
NapAB
Nitrite
NirBD
NrfAH
Ammonia
Assimilatory nitrate reduction
NarB
NR
NosAB
Nit-6
NirA
NasBDE
Denitrification
NarGHI
NirK
NirS
Nitric oxide
NorBC
Nitrous oxide
NosZ
Nitrogen
Nitrogen fixation
NifDKH
AnfG
VnfDKGH
Nitrification
NxrAB
Hao
Hydroxylamine
AmoCAB
Anammox
Hdh
Hydrazine
Hzs

C

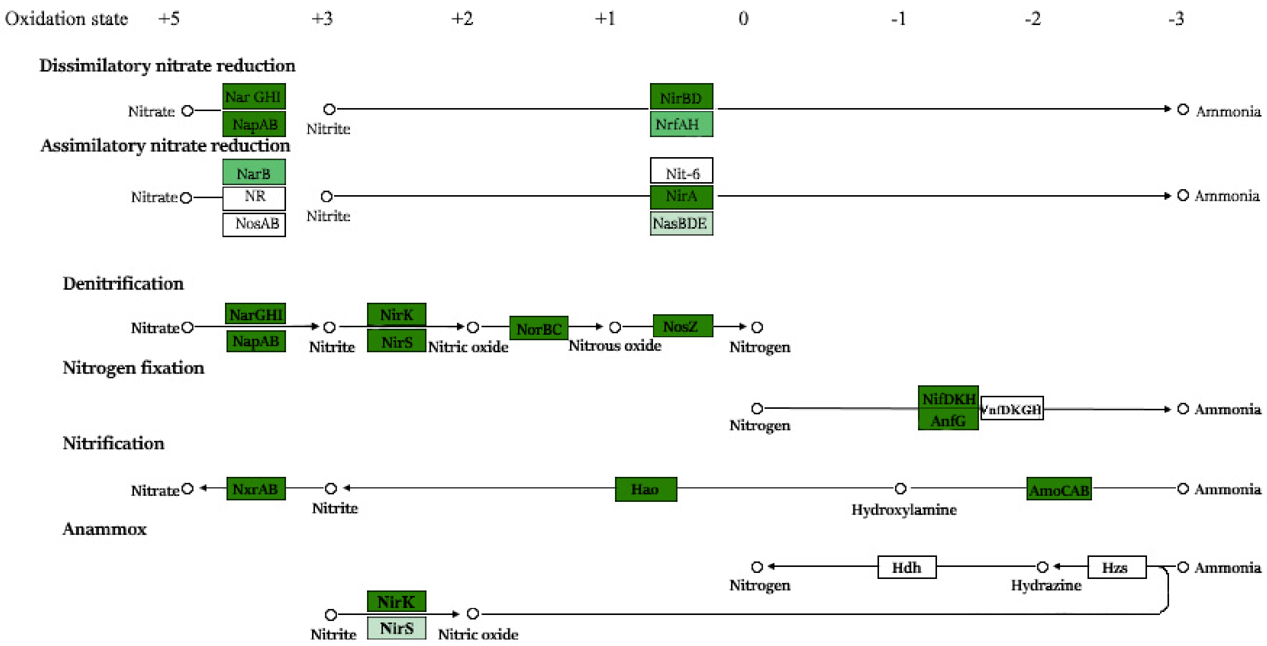

Oxidation state +5 +3 +2 +1 0 -1 -2 -3
Dissimilatory nitrate reduction
Nitrate
Nar GHI
NapAB
Nitrite
NirBD
NrfAH
Ammonia
Assimilatory nitrate reduction
NarB
NR
NosAB
Nit-6
NirA
NasBDE
Denitrification
NarGHI
NirK
NirS
Nitric oxide
NorBC
Nitrous oxide
NosZ
Nitrogen
Nitrogen fixation
NifDKH
AnfG
VnfDKGH
Nitrification
NxrAB
Hao
Hydroxylamine
AmoCAB
Anammox
Hdh
Hydrazine
Hzs

D

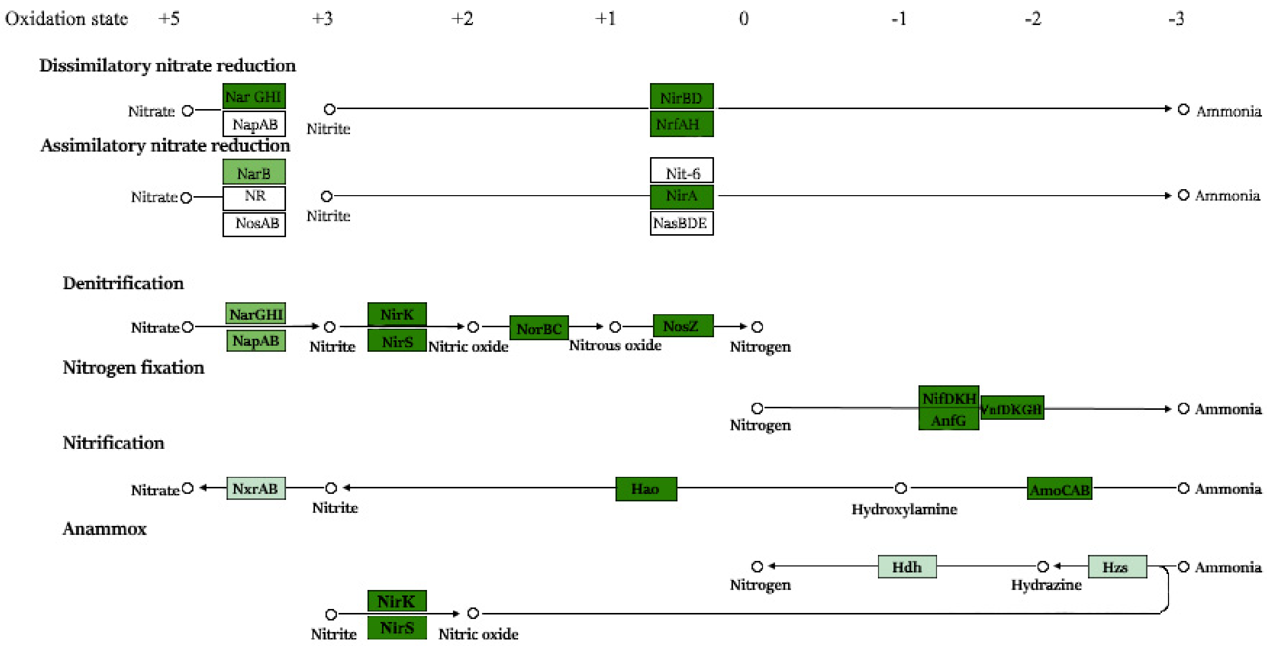

E

Figure S1. Nitrogen metabolic pathways in fed-batch enrichment cultures and continuous-flow cultivated ABR performed by IVICODAK [83]: (A) AOB, (B) NOB, (C) DB, (D) AnAOB, (E) ABR.

Table S1. The number of gene copies of key N cycle bacteria in phase 4 of the ABR operation.

Gene	Number of copies (per 1 mL)
anammox 16S rRNA	(2.41±0.11) x10^{2}
nirS (denitrifiers)	(1.07 ±0.12)x10^{8}
nirK (denitrifiers)	(9.91 ±0.41)x10^{4}
AOB	(1.22±0.37)x10^{8}

Table S2. Bacterial taxon representation in the Venn diagram intersections.

Intersections	Common taxa
A	*Mycobacteriaceae, Chitinophagaceae, Microscillaceae, Crocinitomicaceae, NS9 marine group, Weeksellaceae, NS11-12 marine group, Ignavibacteriaceae, A4b, Babeliaceae, Gemmatimonadaceae, Gemmataceae, Isosphaeraceae, Pirellulaceae, Beijerinckiaceae, Hyphomicrobiaceae, Leptospiraceae, Chthoniobacteraceae, Terrimicrobiaceae, WPS-2*
B	*Flavobacteriaceae, B1-7BS, Methylophilaceae, TRA3-20, Pedosphaeraceae*
C	*Ferroplasmaceae, Ferrovibrionaceae, Oceanibaculaceae*

**D**	*Blastocatellaceae, Streptomycetaceae, Bacteroidaceae, Bacteroidales RF16 group, F082, Prevotellaceae, Rikenellaceae, Nitrososphaeraceae, Gastranaerophilales, Thermaceae Desulfovibrionaceae, Methanobacteriaceae, Acholeplasmataceae, Acidaminococcaceae, Butyricicoccaceae, Christensenellaceae, Erysipelotrichaceae, Ethanoligenenaceae, Hungateiclostridiaceae, Lachnospiraceae, Monoglobaceae, Ruminococcaceae, Halanaerobiaceae, Woesearchaeales, Acetobacteraceae, Acidiferrobacteraceae, Methanomethylophilaceae, Thermoplasmataceae, Petrotogaceae, Akkermansiaceae, Victivallaceae, WCHB1-41*
**E**	*Carnobacteriaceae, Clostridiaceae, Dysgonomonadaceae, Sedimentibacteraceae, Tannerellaceae*
**F**	*Xanthobacteraceae, Burkholderiaceae, Nitrosomonadaceae, Rhodocyclaceae*
**G**	*Comamonadaceae, Rhodanobacteraceae*
**H**	*Alcaligenaceae*
**I**	*Rhizobiaceae*
**J**	*Caulobacteraceae*
**K**	*Pseudomonadaceae*
**L**	*Oscillospiraceae, UCG-010, Sphingomonadaceae*
**M**	*Devosiaceae*
**N**	*Hydrogenophilaceae*

Table S3. Major metabolic pathways in dominant microbial groups (according to KEGG database https://www.genome.jp/)

dominant taxa	Dissimilatory nitrate reduction	Denitrification to N_2	Assimilatory nitrate reduction	Nitrite reduction	N_2 fixation	Anaerobic ammonium oxidation
**Pseudomonadaceae**, g:Pseudomonas	+	+	+	+	-	-
**Rhodanobacteraceae**	NO_2 to NH_4	NO_2 to N_2O	NO_3 to NO_2	-	+	-
**Hydrogenophilaceae**	+	NO_2 to N_2O	NO_3 to NH_4	NH_4 to hydroxylamine	-	-
**Nitrososphaeraceae**	+	NO_2 to NO	+	+	-	-
**Butyricicoccaceae**	-	-	+	-	-	-
**Xanthomonadaceae**, g:Thermomonas, Stenotrophomonas	NO_3 to NO_2	NO_3 to N_2O	+	NO_2 to NO_3	-	-
**Rhizobiaceae**, g:Aminobacter	NO_3	+	+	+	+	-

	to NH_4					
**Sphingomonadaceae**	-	-	NO_3 to NH_4	-	-	-
**Oscillospiraceae**	-	-	-	-	+	-
**Rikenellaceae**	-	-	+	-	-	-
**Bacteroidaceae**	NO_2 to NH_4	-	+	+	-	-
**Alcaligenaceae**, g:Pusillimonas	NO_3 to NH_4	NO_2 to N_2O	NO_3 to NO_2	+	-	-
**Clostridiaceae**	+	-	+	-	+	-
**Carnobacteriaceae**	-	-	+	-	-	-

Note: + means presence, - means absence or unknown
